# Estimating the Prevalence of Cognitive Impairment and Its Associated Factors in Albania: A Nationwide Cross-Sectional Study

**DOI:** 10.3390/brainsci14100955

**Published:** 2024-09-24

**Authors:** Malvina Hoxha, Simonetta Galgani, Jera Kruja, Ilir Alimehmeti, Viktor Rapo, Frenki Çipi, Domenico Tricarico, Bruno Zappacosta

**Affiliations:** 1 Department of the Chemical-Toxicological and Pharmacologic Evaluation of Drugs, Faculty of Pharmacy, Catholic University Our Lady of Good Counsel, 1000 Tirana, Albania; 2 Faculty of Medicine, Catholic University Our Lady of Good Counsel, 1000 Tirana, Albania; gaglasi@tiscali.it; 3 Neurology Service, UHC Mother Teresa, University of Medicine, Tirana, 1005 Tirana, Albania; jkruja@gmail.com; 4 Department of Family and Occupational Health, Faculty of Medicine, University of Medicine, Tirana, 1005 Tirana, Albania; iliralimehmeti@gmail.com; 5 Health Commission, Academy of Sciences of Albania, 1000 Tirana, Albania; 6 Faculty of Pharmacy, Catholic University Our Lady of Good Counsel, 1000 Tirana, Albania; v.rapo6451@stud.unizkm.al (V.R.); f.cipi7890@stud.unizkm.al (F.Ç.); 7 Department of Pharmacy-Pharmaceutical Sciences, Faculty of Pharmacy, University of Bari “Aldo Moro”, 70121 Bari, Italy; domenico.tricarico@uniba.it; 8 Department of Biomedical Sciences, Faculty of Medicine, Catholic University Our Lady of Good Counsel, 1000 Tirana, Albania; b.zappacosta@unizkm.al

**Keywords:** cognitive impairment, dementia, Albania, MMSE, Early Dementia Questionnaire

## Abstract

Background/Objectives: Cognitive impairment is an intermediate state between normal aging and dementia, and its detection in the early stages is essential to prevent dementia, an incurable pathology. The aim of this study is to screen and estimate the prevalence of cognitive impairment, including dementia, and its correlated factors in a community-based sample of the Albanian population over 50 years old. Methods: We carried out a door-to-door neuropsychological screening of Albanian residents older than 50 years from November 2023 to June 2024 in 12 Albanian districts. Participants completed the Early Dementia Questionnaire (EDQ) and the Mini Mental State Examination (MMSE). Results: The overall estimating prevalence of cognitive impairment and early dementia among the Albanian population over 50 years old was 14.04% using the MMSE, with 2.31% for MMSE ≤18 (serious cognitive impairment), 5.51% for MMSE 19–22 (mild cognitive impairment (MCI)), and 6.22 for MMSE 23–24 (suspected cognitive impairment or dementia),respectively. The prevalence of early dementia using the EDQ was significantly higher (53.99%).The number of male participants with MMSE scores of 23–24 (suspected cognitive impairment or early dementia) was 2.5 times higher with respect to female participants. Smoking and alcohol consumption decreased the MMSE scores. The number of participants with normal cognitive function (MMSE scores 25–30) was lower among participants with hypertension, diabetes, hyperlipidemia, and cerebral ischemia. Conclusions: A diagnostic evaluation, including a clinical examination, neuroimaging, and laboratory studies, is further required for a diagnosis. Despite limitations, the data provided in this study are the only ones reported for a large community-based sample of the older adult Albanian population, which can help health care providers to diagnose cognitive impairment.

## 1. Introduction

### 1.1. Background/Rationale

Cognitive impairment (CI) is a major risk factor for the development of dementia, and early pharmacologic treatment of subjects with cognitive impairment without dementia (CIND), commonly named mild cognitive impairment (MCI), may delay progression to dementia [1]. Considering that cognitive impairment is often unrecognized, and that the aging population is increasing, the number of patients with mild cognitive impairment and dementia is expected to increase. Different approaches are reported to slow cognitive decline, such as exercise, the Mediterranean diet, and treatment of cardiovascular risk factors [2].

The global prevalence of MCI is reported to be 15.56%. According to the World Health Organization (WHO), currently, approximately 50 million people worldwide live with a severe form of cognitive impairment, 60% of whom come from low-and middle-income countries [3]. In addition, the prevalence of dementia is expected to triple in 2050 [4].

According to the Institute of Statistics of Albania (INSTAT), in January 2022, the Albanian population was reported to be 2.79 million persons [5]. Except during the COVID-19 pandemic period (2020–2021), the life expectancy at birth in Albania has been increasing for years, with women tending to live 4.3 years longer than Albanian males [5]. According to the World Health Organization (WHO), life expectancy was reported to be 78 years in 2020; 76.3 years for females versus 76.3 for males [6]. 

In Albania, there are very few epidemiological studies on cognitive impairment, which were carried out in only two cities (Tirana and Shkodër). The most recent study, published in 2020, showed a prevalence of mild cognitive impairment of 19.42% among 206 participants (average age 68.8 years) in Tirana and Shkodër [7]. Previously, in 2012, Kruja et al. showed a dementia prevalence of 9.6% in two Albanian cities (Tirana and Saranda) [8]. This percentage was increased compared to the prevalence in 1999 (7.75%; 11.45% women vs. 4.83% men) [9].

### 1.2. Objectives

The aim of this study was to screen and estimate the prevalence of cognitive impairment and early dementia in a community-based sample of the Albanian population, including all districts of the country, and to study the associated effects of lifestyle, socio-demographic, and clinical factors.

## 2. Methods

### 2.1. Study Design and Setting

A community cohort study was performed in Albania, in 12 counties of the country, including Tirana, Vlorë, Shkodër, Lezhë, Gjirokastër, Fier, Elbasan, Korcë, Durrës, Dibër, Berat, and Kukës, respectively. The population for this study was randomly chosen, and a door-to-door neuropsychological screening was performed from November 2023 to June 2024. 

Residents of Albania aged 50 years or older were screened. Considering that, in Albania, no study has ever been carried out on residents 50–59 years old, we also assessed if lifestyle and comorbidities may influence the prevalence of cognitive impairment even in this group of participants.

The screening of residents of Albania older than 50 years consisted of two phases: the completion of a socio-demographic and health assessment questionnaire (Appendix A) and neuropsychological screening tools, including the Mini Mental State Examination (MMSE) and the Early Dementia Questionnaire (EDQ). The MMSE was used as the “gold standard” screening tool for cognitive impairment and dementia. The use of the EDQ in our study was influenced by its high sensitivity in the early stages of cognitive impairment/dementia and non-influence by education and cultural level.

Three authors (M.H., F.Ç., and V.R.) administered the questionnaire and the MMSE/EDQ to subjects that fulfilled the inclusion criteria. All participants provided informed consent.

### 2.2. Participants

The criteria for eligibility were as follows: Albanian population, resident in Albania aged 50 years or older, capable and willing to complete the questionnaire and the MMSE/EDQ. Non-Albanian citizens, participants younger than 50 years old, Albanian citizens not residing in Albania, patients with an already known diagnosis of dementia, or who were followed in specialist structure for cognitive impairment were excluded from the study. 

### 2.3. Variables

We collected data on the participants’ age, gender, residence, education, civil status, living arrangements, employment, smoking and alcohol consumption, physical activity, chronic disease, hypertension, diabetes, hyperlipidemia, cerebral ischemia, antidepressant use, infectious disease, other neurological diseases in the family, family history of cognitive impairment, and/or dementia.

### 2.4. Data Sources/Measurement

Through the health assessment questionnaire, we collected data on participants’ comorbidities and lifestyle. The MMSE and EDQ were used as neuropsychological screening tools.

### 2.5. MMSE

We used the Mini Mental State Examination as a screening tool for cognitive impairment and dementia, which assesses memory (3 points), orientation (5 points), recall and calculation (5 points), language (9 points), and registration (3 points) [10,11,12,13]. Different studies report various cut-off values of the MMSE for early dementia for different populations [14]. The MMSE was previously translated and validated in Albania by the Neurology Service, University of Medicine, Tirana, Albania, but there is not any publication in scientific journals in this regard, except an abstract in the proceedings of the 6th Congress of the European Federation of Neurological Societies (Vienna 26–29 October 2002) [9]. The lack of published data can negatively impact conclusions and limit statistical analysis. However, in our study, despite the lack of published data on the field, we displayed the necessary information and used two neurological screening tools for cognitive decline and early dementia. The MMSE consists of 30 questions, corresponding to a total of 30 points, administered in 10 min. A cut-off of 23/24 indicates subjects with suspected cognitive impairment, a cut-off of 18–22 indicates MCI, a cut-off of 18 or lower indicates serious cognitive impairment, and a cut-off of 25–30 indicates normal cognitive functions [14].

### 2.6. Early Dementia Questionnaire

The Early Dementia Questionnaire [15,16] was translated in Albanian and validated by three of the authors (M.H., B.Z., and S.G.). The scores vary from 0 corresponding to never, to 1 for seldom, 2 for sometimes, and 3 for always. The scores are based on the symptoms that the patient experienced in a week for the past 2 years. The maximum score is 60, and the minimum score is 0. A cut-off score of 8 or more indicates that the patient has possible early dementia [15]. The dementia symptoms are classified in 6 groups, respectively: memory, concentration, symptoms, physical symptoms, emotions, sleep, and others. The memory symptoms consist of 5 questions, the concentration symptoms consist of 4 questions, the physical symptoms consist of three questions, the emotional symptoms consist of 4 questions, the sleep disturbance symptoms consist of two questions, and other symptoms consist of two questions. Validation of the questionnaire was performed with 15 participants who fulfilled the inclusion criteria (Appendix B). The required answers to the EDQ questions could also be provided by the informant; however, in our study, we did not have any case, hence the “informant” did not have any impact on the results in our study. 

The cut-off score of the EDQ used for early dementia, also reported in another study, was 8 [15]. However the validity of the EDQ was not previously assessed in Albania, and there was no possibility of comparison with previous studies.

### 2.7. Bias

Some of the sources of bias of this study are as follows: the sampling bias, the recall bias, and the interviewer bias. In order to address the interviewer and sampling bias as potential sources of bias, three of the authors who interviewed the participants met in advance, before interviewing the study participants, and discussed and clarified all doubts related to the questions provided to subjects and the sampling methods.

### 2.8. Study Size

**Figure 1:**
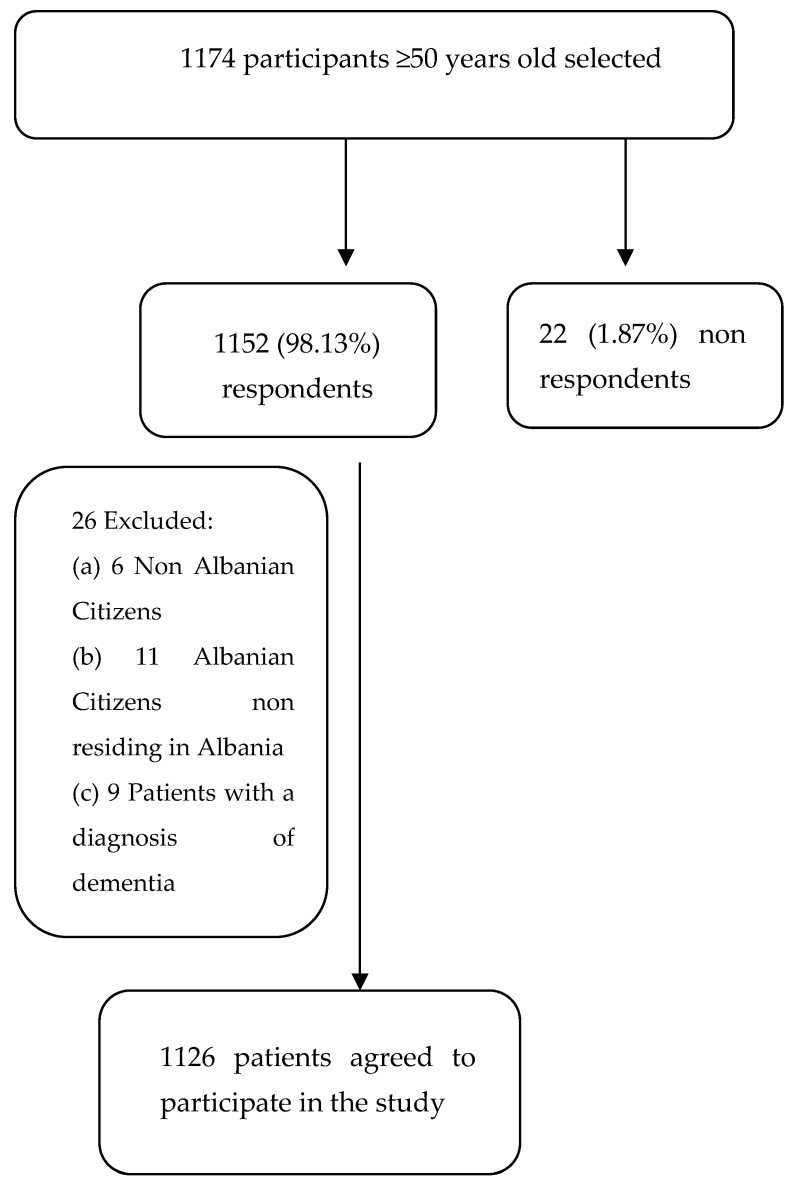
Flowchart of the inclusion of participants in the study.

We identified a total of 1174 Albanian participants of ≥50 years old, among whom 22 refused to respond (Figure 1).

We excluded 26 subjects from the study for the following reasons: non-Albanian citizens (6 subjects), Albanian citizens non residing in Albania (11 subjects), and individuals with a diagnosis of dementia (9). Thus, 1126 subjects agreed and were enrolled in the study (Figure 1).

### 2.9. Quantitative Variables

Lifestyle and comorbidity variables were analyzed after classifying them in groups according to participants with either MMSE scores (MMSE 18 or lower; 18–22; 23/24, 25–30), or EDQ scores (lower than 8, and greater or equal to 8).

### 2.10. Statistical Methods

Descriptive statistic analyses (mean, standard deviation, and percentage) were used to assess the demographic variables. Data were analyzed using R software, version 4.2.0 for Windows©2009 (Cary, NC, USA). A *p*-value of less than 0.05 was considered as statistically significant. The Chi-square test was used to assess the association of dementia with other variables for the EDQ.

### 2.11. Ethics Approval

The study was performed in accordance with the Declaration of Helsinki and approved by the Ethics Committee of the Catholic University “Our Lady of Good Counsel” (date 16 November 2023; no.528). All patients were informed on the objective of the study and on data protection according to art. 5 GDPREU/2016/679, as briefly reported below: a pseudonymization protocol is applied to mask personal data that are not used for other purposes different from that described in the informed consent and in the manuscript. Neither personal nor pseudonymized data will be disseminated. In the event that the results of the study are disseminated, only aggregated and anonymous data will be used, for instance, in the case of this publication. When possible, participants provided informed consent.

## 3. Results

A total of 1126 people over 50 years old participated in the study, including 894 (79.39%) from Tirana, and 232 participants (20.60%) from other districts of Albania, specifically, Vlorë, Durrës, Berat, Elbasan, Fier, Korçë, Has, Kukës, Laç, Libohovë, Mirditë, Patos, Përmet, and Shkodër. All participants were interviewed face to face.

The majority of the respondents were female (60.92%) (Table 1). The number of participants decreased with age, ranging from 22.02% for the age group of 50–54 years old, to 2.31% for the age group of 85+ years old. A total of 86.15% of the patients were married, while 10.48% were widowed, 1.6% were single, and 1.24% were divorced (Table 1). The socio-demographic characteristics of the Albanian participants over 50 years old are reported in Table 1.

The median age of participants was 62 years old. A total of 95.03% lived with family, while 4.62% lived alone. Additionally, 47.42% of participants were employed versus 43.69% who were retired. The most frequent level of education was high school studies (46%) followed by university studies (27.53%), and middle school studies (18.47%). Only 1.06% of the respondents had no formal education (Table 1).

The mean MMSE score for all participants was 27.51 (27.21–27.81; *p*-value < 0.001). As suggested by Arevalo et al., in our study, we used an MMSE cut off of 23–24 to detect individuals with suspected cognitive impairment [11] and a cut-off score of the EDQ for early dementia of 8 [15]. 

Among the 1126 Albanian residents over 50 years old who were screened, 14.04% were positive for cognitive impairment using the MMSE, with 2.31% for MMSE ≤18 (serious cognitive impairment), 5.51% for MMSE 19–22 (mild cognitive impairment (MCI)), and 6.22 for MMSE 23–24 (suspected cognitive impairment), respectively. The prevalence of early dementia using the Early Dementia Questionnaire was significantly higher (53.99%) with respect to the MMSE, in line with other studies that have shown a higher prevalence of dementia using both the MMSE and EDQ (52.33% using the MMSE and 15.3% using the EDQ, respectively) [15].

The correlation between cognitive decline and aging is widely demonstrated in different studies. The data in our study showed that the prevalence of MCI increased from 0.35% to 1.30% for ages 55–59 years to 60–64 years, respectively. In addition, the results indicated that the number of patients with normal cognitive function (MMSE scores 25–30) decreased with age: 18.82% for ages 50–54 years, 18.65% for ages 55–59 years, 11.37% for ages 60–64 years, 11.30% for ages 70–74 years, 7.81% for ages 75–80, 4.79% for ages 81–85 years, and 2.31% for ages 85+ years (Table 2). The same trend was also demonstrated using the EDQ, where the number of patients with normal EDQ scores decreased with age: 46%for ages 50–54 years, 9.24% for ages 55–59 years, 7.10% for ages 70–74 years, 3.91% for ages 75–80, 3.19% for ages 81–85 years, and 0.79% for ages 85+ years (Table 2).

The number of male participants with MMSE scores of 23–24 (suspected cognitive impairment) was 2.5 times higher with respect to female participants. The number of females with normal MMSE and EDQ scores was higher with respect to males (55.42% females versus 30.55% males for MMSE 25–30, and 33.13% females versus 20.96% males for EDQ scores ≥8).

The number of participants with possible dementia increased in the capital city and also in other cities of Albania using the Early Dementia Questionnaire. The participants who were married had a higher prevalence of cognitive impairment (12.61%) and potential dementia (45.47%) with respect to participants who were single (0.35% and 0.18% respectively), divorced (0.18% and 0.89%)or widowed (0.88% and 6.66%), using both the MMSE and EDQ (Table 2). As expected, for the EDQ, age, education, residence, living arrangement, and employment were associated with potential dementia, in contrast to gender and civil status (*p* > 0.05) (Table 2).

In the lifestyle and clinical characteristic section, the participants were queried about their smoking and drinking habits. Table 3 reports the number of participants in each group. According to the MMSE scores, there were 44 (3.91%) smokers with MMSE scores ≤24 versus 96 (8.53%) non-smoker participants, and 16 former smoker participants with the same MMSE scores. The number of non-smoker participants with normal MMSE scores (25–30) was significantly higher (762, 67.67%) with respect to smoker participants with the same MMSE scores (108, 9.59%). Considering that alcohol can be one of the causes of dementia, the number of participants with normal MMSE scores (25–30) who never consumed alcohol was higher (612, 54.35%) with respect to participants who had 1–2 drinks/day, more than 2 drinks/day, several times a week, or rarely. Increasing the amount of alcohol consumption reduced the number of participants with normal MMSE scores. No similar trend was found for the EDQ (Table 3).

The number of participants with normal EDQ scores increased with physical activity: 226 (20.07%) for participants with 5–7 times of physical activity/week, versus 152 (13.49%) for 1–4 times/week, and 137 (12.17%) for no physical activity. No statistical significance was found for cognitive impairment and physical activity using the MMSE (Table 3).

Three hundred and eighty three participants with chronic disease (34.01%) had potential dementia using the cut-off of 8 or more for the EDQ, versus 285 participants with chronic disease (25.31%) with a cut-off of less than 8 for the EDQ (Table 3). No statistical significance was found for the association between chronic disease and cognitive impairment/dementia using the MMSE. The prevalence of potential dementia using the EDQ as a screening tool was higher in participants with infectious disease (427, 37.92%) with respect to participants with no infectious disease (179, 15.89%).

The number of participants with normal cognitive function (MMSE scores 25–30) was lower in participants with hypertension (444, 39.43%) with respect to participants who did not suffer from essential hypertension (521, 46.27%) (Table 3). The same trend was also showed for participants with diabetes (148, 13.14%) versus participants without diabetes (808, 71.76%); for participants with hyperlipidemia (276, 24.51%) versus participants with no hyperlipidemia (692, 61.46%); and for participants with cerebral ischemia (30, 2.66%) versus participants with no cerebral ischemia (928, 82.41%) (Table 3).

When asked about the use of antidepressants, the results showed that the prevalence of potential dementia using the EDQ was higher among participants who did not use antidepressants (519, 46.09%) with respect to participants who took the drugs (92, 8.17%). In line with this, the number of participants with normal cognitive function (MMSE scores 25–30) was higher in participants with no use of antidepressants (868, 77.0%) with respect to those who used them (97, 8.61%) (Table 3). The percentage of participants with normal cognitive function increased in participants with no other neurological diseases in the family (72.20% versus 13.49%), or family history of cognitive impairment and/or dementia (71.22% versus 14.29%), respectively (Table 3).

Physical activity, chronic disease, hypertension, diabetes, hyperlipidemia, cerebral ischemia, antidepressants, family history of cognitive impairment and/or dementia, and neurological diseases were associated with potential dementia using the EDQ (Table 3).

## 4. Discussion

The number of people with cognitive impairment and dementia is increasing with aging worldwide, and often distress and disability go unrecognized [17]. The population in Albania is around 2,761,785 inhabitants, among whom 1,017,926 are aged 50 years and older [18]. Tirana, the main district of Albania, is the most populated, followed by the counties of Durrës and Fier, with 10.5% and 9.8% of the population, respectively [18].

Different studies have been carried out “door-to-door” using the MMSE as a screening method to estimate the prevalence of cognitive defects [12,19]. In this study, we screened 1126 people over 50 years old, using two screening tools, the MMSE and EDQ, respectively. Only 2.31% of the participants were positive for MMSE ≤18 (serious cognitive impairment), 5.51% for MMSE 19–22 (mild cognitive impairment (MCI)), and 6.22 for MMSE 23–24 (suspected cognitive impairment or dementia) using the MMSE. The prevalence of possible early dementia using the EDQ was significantly higher (53.99%). Individuals scoring low in MMSE screening were recommended a complete neurological examination. The differences in prevalence through the MMSE and EDQ are also reported in other studies that indicate a prevalence for early dementia almost 3.5 times higher for the EDQ with respect to the MMSE [15]. This difference could be attributed to the fact that many questions in the EDQ assess very early symptoms of dementia, not excluding mild cognitive impairment symptoms, and other cognitive impairments that may bring a high false positive rate. The MMSE is not effective in assessing cognitive function decline in the early course of dementia. Considering also that respondents should have a certain level of education and knowledge of calculation, this would contribute to the differences in the prevalence of dementia assessed by both tests. In both cases, a neurological visit should be undertaken to determine the diagnosis.

Earlier, Kruja et al. reported a prevalence of dementia of 7.75% for Albanian residents in 1999 aged ≥60 years [9]. The prevalence of dementia was more than twice as high in females with respect to males (11.45% vs. 4.83%) and almost tripled among residents in the 65–69-years-old group (6.27%) compared to younger participants in the 60–64-years-old group (2.07%) [9]. As previously reported in other study, the prevalence of MCI in two cities of Albania, Shkoder and Tirana, was 75.73 and 20.39% using the MoCA/MoCA B and Mini-Cog scales, respectively [7]. In this study, we also used the EDQ, which is a very sensitive neurological screening tool used for earlier detection ofcognitive impairment. For this reason, we also included a representative sample of the population of 50–60 years old for evaluating if lifestyle and comorbidities may influence the prevalence of cognitive impairment even in this group of participants.

As expected, the number of patients with normal cognitive function (MMSE scores 25–30) decreased with age. The number of male participants with MMSE scores of 23–24 (suspected cognitive impairment) was 2.5 times higher with respect to female participants. The participants who were married had a higher prevalence of cognitive impairment (12.61%) and potential dementia (45.47%) with respect to participants who were single (0.35% and 0.18% respectively), divorced (0.18% and 0.89%),or widowed (0.88% and 6.66%) using both the MMSE and EDQ. Smoking and alcohol consumption decreased cognitive function and the MMSE scores. The number of participants with normal EDQ scores increased with physical activity. The number of participants with normal cognitive function (MMSE scores 25–30) was lower among participants with hypertension, diabetes, hyperlipidemia, and cerebral ischemia. The potential dementia risk increased in participants with chronic disease using the EDQ as a screening tool.

The strength of our study is the use of two assessment tools for cognitive decline and early dementia. In the case of the EDQ, a potential strength is the non complete dependence on the information from the patient, considering their distraction and forgetfulness. The required answers to the questions could also be provided by the informant [15]. The EDQ is also quick, simple, easily administered to participants, and not influenced by education and cultural level. In addition, another strength of our study is the high response rate, which probably could be attributed to the type of approach the interviewer used with the study participants in easily explaining to the subjects the meaning of cognitive defects, the importance for their health, and the importance of the study, which could have cultivated overall curiosity in the field. In addition, the lack of public media information on cognitive defects may also have increased the study participation rate.

A limitation of this study is that a clinical examination, including neuroimaging and laboratory tests, should be performed to diagnose cognitive impairment and dementia. The number of participants over 65 years old should be increased in further studies. Despite the limitations and controversies on the EDQ cut-off score, a score above 8 can inform physicians of potential cognitive decline and further examinations can be carried out. In addition, the MMSE also has some limitations, such as the influence of education and cultural level, and lack of sensitivity for early stages of cognitive impairment. Considering that the MMSE changes over time, participants should be monitored continuously instead of a single measurement. Recall bias is also another limitation of the study, considering that participants provided retrospective data.

Another limitation is that other screening tools, such as the Informant Questionnaire on Cognitive Decline in the Elderly (IQCODE), should have been used. In addition, as suggested by Salzman (2012), the use of benzodiazepines may be related to impaired cognition in a dose-dependent manner [20]; hence, another limitation of this study is the lack of information on benzodiazepine use in our study sample.

In addition, considering that this study is a cross-sectional study, the information provided is a snapshot of the current situation, in contrast with longitudinal studies where usually changes in cognitive function are assessed. Sampling bias, sometimes picked by convenience, is another limitation of the study.

The” door-to-door” approach has also some limitations that can lead to information bias, for example, the lack of interest of participants in providing all of the detailed information and additional data that could help in analysis. Despite the participants providing informed consent and also being informed in detail about the study, with the official contact details of the study organizers, skepticism related to the interviewer, to the scope of the study, or any potential political involvement are some of the sources of information bias. The participants’ level of education also influences the truthfulness of the answers. We noticed that participants with a low level of education were not varied in their responses, indicating almost every time “never” as a response instead of “seldom,” “sometimes,” or “always.” The fear or complexity of answering certain questions related to mental health, such as “the use of antidepressants,” “presence of cognitive impairment in family,” and “other neurologic diseases,” was also present in many participants. Concealment of the truth related to personal data, such as name, civil status, and education, could also have influenced the results. Interviewer bias could also have influenced the results.

## 5. Conclusions

This study contributes to filling the gap of knowledge on the current epidemiologic situation of cognitive impairment and dementia in Albania. Considering that cognitive impairment and dementia are underdiagnosed, the screening of vulnerable individuals is important for further examination and for taking preventative steps toward coping with this situation. Despite limitations, the data provided in this study are the only ones reported for a large community-based sample of older adults in the Albanian population, which can help health care providers to diagnose cognitive impairment and dementia but also serve as a guideline for policymakers and social services to improve the quality of life of patients and their caregivers.

## Figures and Tables

**Table 1:** Socio-demographic characteristics of the Albanian participants over 50 years old.

Variables	Number → n (%)	Percentage %
Gender		
Female	686	60.92
Male	440	39.08
Age range		
50–54	248	22.02
55–59	232	20.60
60–64	164	14.56
65–69	150	13.32
70–74	140	12.43
75–79	100	8.88
80–85	66	5.86
85+	26	2.31
Residence		
District of Tirana	894	79.39
Other Albanian Districts	232	20.60
Education		
No education	12	1.06
Elementary school	56	4.97
Middle school	208	18.47
High school	518	46.00
University	310	27.53
PhD, Prof	18	1.6
Civil status		
Single	18	1.6
Married	970	86.15
Divorced	14	1.24
Widow	118	10.48
Living Arrangements		
Alone	52	4.62
With family	1070	95.03
With friends	0	0
Employment		
Employed	534	47.42
Unemployed	112	9.95
Retired	492	43.69

**Table 2:** Association of MMSE and EDQ scores with participants’ socio-demographic characteristics.

	MMSE ≤ 18 n (%)	MMSE 19–22 n (%)	MMSE 23–24 n (%)	MMSE 25–30 n (%)	EDQ < 8 n (%)	EDQ ≥ 8 n (%)	X^2^	*p*-Value
Total	26 (2.31)	62 (5.51)	70 (6.22)	968 (85.97)	518 (46.00)	608 (53.99)		
Age range							12.37	0.015
50–59	4 (0.35)	28 (2.49)	24 (2.12)	422 (37.47)	220(19.54)	258 (22.91)		
60–69	16 (1.42)	18 (1.48)	20 (1.78)	258 (22.91)	124 (11.01)	188 (16.7)		
70–74	2 (0.18)	12 (1.06)	10 (0.89)	116 (10.30)	80 (7.10)	60 (5.33)		
75–79	2 (0.18)	0 (0.00)	10 (0.89)	88 (7.81)	44 (3.91)	56 (4.97)		
80–85+	2 (0.18)	4 (0.35)	6 (0.53)	80 (7.10)	45 (3.98)	47 (4.18)		
Gender							0.06	0.81
Female	10 (0.89)	32(2.84)	20 (1.78)	624 (55.42)	313 (27.79)	373 (33.13)		
Male	16 (1.42)	30 (2.66)	50 (4.44)	344 (30.55)	204 (18.12)	236 (20.96)		
Residence							5.27	0.02
Capital City	20 (1.78)	54 (4.79)	62 (5.51)	758 (67.32)	426 (37.83)	468 (41.56)		
Other Cities	7 (0.62)	8 (0.71)	8 (0.71)	209 (18.56)	91 (16.16)	141 (12.52)		
Education							62.83	0.00
No education	0 (0.00)	0 (0.00)	2 (0.18)	10(0.89)	1 (0.09)	11 (0.97)		
Elementary school	0 (0.00)	6 (0.53)	6 (0.53)	44 (3.91)	5 (0.44)	51 (4.53)		
Middle school	0 (0.00)	6 (0.53)	10 (0.89)	192 (17.05)	248 (22.02)	270 (23.98)		
High school	20 (1.78)	26 (2.31)	26 (2.31)	447 (39.69)	73 (6.48)	135 (11.99)		
University, PhD	6 (0.53)	26 (2.31)	26 (2.31)	269 (23.89)	185 (16.43)	143 (12.69)		
Civil status							6.64	0.09
Single	0 (0.00)	0 (0.00)	4 (0.35)	13 (1.15)	8 (0.71)	10 (0.88)		
Married	24 (2.13)	58 (5.15)	60 (5.33)	829 (73.62)	458 (40.67)	512 (45.47)		
Divorced	0 (0.00)	0 (0.00)	2(0.18)	12 (1.06)	4 (0.35)	10 (0.89)		
Widow	2(0.18)	4 (0.35)	4 (0.35)	108 (9.59)	43 (3.82)	75 (6.66)		
Living Arrangements							4.97	0.03
Alone	0 (0.00)	2 (0.18)	4 (0.35)	45 (3.99)	16 (1.42)	36 (3.19)		
With family	26 (2.31)	60 (5.33)	66(5.86)	919 (81.62)	498 (44.23)	572 (50.79)		
Employment							18.43	0.00
Employed	12 (1.06)	32 (2.84)	36 (3.19)	455 (40.41)	280 (24.87)	254 (22.56)		
Unemployed	4 (0.35)	8 (0.71)	8 (0.71)	92 (8.17)	44 (3.91)	68 (6.04)		
Retired	10 (0.89)	24 (2.13)	28 (2.49)	429 (38.09)	196 (17.41)	296 (26.29)		

**Table 3:** Association of MMSE and EDQ scores with participants’ lifestyle and clinical characteristics.

	MMSE ≤ 18 n (%)	MMSE 19–22 n (%)	MMSE 23–24 n (%)	MMSE 25–30 n (%)	EDQ < 8 n (%)	EDQ ≥ 8 n (%)	X^2^	*p*-Value
Total	26(2.31)	62(5.51)	70(6.22)	968(85.97)	518(46.00)	608(53.99)		
Age range							12.37	0.015
50–59	4 (0.35)	28 (2.49)	24 (2.12)	422 (37.47)	220(19.54)	258 (22.91)		
60–69	16 (1.42)	18 (1.48)	20 (1.78)	258 (22.91)	124 (11.01)	188 (16.7)		
70–74	2 (0.18)	12 (1.06)	10 (0.89)	116 (10.30)	80 (7.10)	60 (5.33)		
75–79	2 (0.18)	0 (0.00)	10 (0.89)	88 (7.81)	44 (3.91)	56 (4.97)		
80–85+	2 (0.18)	4 (0.35)	6 (0.53)	80 (7.10)	45 (3.98)	47 (4.18)		
Gender							0.06	0.81
Female	10 (0.89)	32(2.84)	20 (1.78)	624 (55.42)	313 (27.79)	373 (33.13)		
Male	16 (1.42)	30 (2.66)	50 (4.44)	344 (30.55)	204 (18.12)	236 (20.96)		
Smoking							4.82	0.09
Smoker	8 (0.71)	12(1.06)	24 (2.13)	108 (9.59)	57 (5.06)	95 (8.44)		
Non-smoker	18 (1.59)	44 (3.91)	34 (3.02)	762 (67.67)	403 (35.79)	455 (40.41)		
Former smoker	0 (0.00)	4 (0.35)	12(1.07)	94 (8.35)	48 (4.26)	62 (5.51)		
Consumption of alcohol							1.79	0.77
Never	14 (1.24)	32 (2.84)	26 (2.31)	612(54.35)	316 (28.06)	368 (32.68)		
1–2 drinks a day	4 (0.35)	8 (0.71)	0(0.00)	36 (3.19)	28 (2.48)	26 (2.31)		
>2 drinks a day	0 (0.00)	2 (0.18)	0(0.00)	18 (1.59)	8 (0.71)	12 (23.98)		
Several times a week	0 (0.00)	6 (0.53)	10 (0.89)	80(7.10)	40 (3.55)	56 (4.93)		
Rarely	8 (0.71)	14 (1.24)	28 (2.49)	228 (20.25)	126 (11.19)	152(13.49)		
Physical activity, how many times per week							14.59	0.00
Never	4 (0.35)	24 (2.13)	19 (1.69)	301 (26.73)	137(12.17)	211 (18.74)		
1–4	10 (0.89)	18 (1.59)	18(1.59)	298 (26.46)	152 (13.49)	192 (17.05)		
5–7	10 (0.89)	20 (1.78)	34 (3.02)	364 (32.33)	226 (20.07)	202(17.94)		
Chronic Diseases							6.54	0.01
Yes	14 (1.24)	28 (2.49)	42 (3.73)	584 (51.86)	285 (25.31)	383 (34.01)		
No	12 (1.0)	35 (3.11)	28(2.49)	373 (33.13)	226 (20.07)	222 (19.71)		
Hypertension							11.82	0.00
Yes	14 (1.24)	24 (2.13)	34 (3.02)	444 (39.43)	206 (18.29)	310 (27.53)		
No	14 (1.24)	39 (3.46)	36 (3.19)	521 (46.27)	306 (27.17)	304 (26.99)		
Diabetes							6.86	0.01
Yes	2 (0.18)	14 (1.24)	10 (0.89)	148 (13.14)	64 (5.68)	110 (9.77)		
No	24 (2.13)	48 (4.26)	60 (5.33)	808 (71.76)	447 (39.69)	493 (43.78)		
Hyperlipidemia							12.41	0.00
Yes	4 (0.35)	32 (2.84)	20 (1.78)	276 (24.51)	125 (11.10)	207 (18.38)		
No	22 (1.95)	30 (2.66)	50 (4.44)	692 (61.46)	390 (34.63)	404 (35.88)		
Cerebral ischemia							9.83	0.00
Yes	0 (0.00)	2 (0.18)	0 (0.00)	30 (2.66)	6 (0.53)	26 (2.31)		
No	26 (2.31)	60 (5.33)	70 (6.22)	928 (82.41)	507 (45.03)	577 (51.24)		
Antidepressants							45.76	0.00
Yes	0 (0.00)	0 (0.00)	11 (0.98)	97 (8.61)	16 (1.42)	92 (8.17)		
No	27 (2.39)	61 (5.42)	60 (5.33)	868 (77.09)	497 (44.14)	519 (46.09)		
Infectious diseases							1.23	0.27
Yes	16 (1.42)	48 (4.26)	46 (4.08)	662 (58.79)	345 (30.64)	427 (37.92)		
No	10 (0.89)	14 (1.24)	24 (2.13)	298 (26.46)	167 (14.83)	179 (15.89)		
Other neurological diseases in the family							17.91	0.00
Yes	0 (0.00)	4 (0.35)	18 (1.59)	152 (13.49)	54 (4.79)	120 (10.66)		
No	26 (2.31)	58 (5.15)	53 (4.71)	813 (72.20)	460 (40.85)	490 (43.52)		
Family history of cognitive impairment and/or dementia							8.77	0.00
Yes	0 (0.00)	8 (0.71)	11 (0.98)	161 (14.29)	64 (5.68)	116 (10.30)		
No	26 (2.31)	54 (4.79)	60 (5.33)	802 (71.22)	448 (39.79)	494 (43.87)		
Diagnosis							13.73	0.00
Physician	0 (0.00)	4 (0.35)	0 (0.00)	26 (2.31)	20 (1.78)	10 (0.89)		
Neurologist and/or geriatrician	0 (0.00)	2 (0.18)	10 (0.89)	88 (7.81)	32 (2.84)	68 (6.04)		
Hospital structure	6 (0.53)	4 (0.35)	12 (1.06)	50 (4.44)	22 (1.95)	50 (4.44)		

## Data Availability

Due to privacy, legal, and ethical reasons, the data that support the findings of this study are available upon reasonable request to support projects with the potential for patient benefit.

## Appendix A

QUESTIONNAIRE

I. Participant Information
  Name Surname
  Address
  Telephone number
II. Patient companion information (if applicable)
  Name Surname
  Relationship with the patient
  Telephone number
III. Socio-demographic data
  1. Gender □ F □M
  2. Age
  3. Residence (City/Village where you live)
  4. Civil status
  □ Single
  □ Married
  □ Divorced
  □ Widow
  5. Living Arrangements
  □ Alone
  □ With family
  □ With friends
  □ None of the above
  □ Specify
  6. Education
  □ No education
  □ Elementary school
  □ Middle school
  □ High school
  □ University
  □ PhD, Prof
  7. Employment
  □ Employed
  □ Unemployed
  □ Retired
  8. Smoking
  □ Smoker
  □ Non-smoker
  □ Former smoker
  9. Consumption of alcohol
  □ Never
  □ 1–2 drinks a day
  □ >2 drinks a day
  □ Several times a week
  □ Rarely
IV. Clinical data
  10. Physical activity, how many times a week
  □ Never
  □ 1–4
  □ 5–7
  11. Do you suffer or have you suffered from chronic disease/s?
  □ Yes
  □ No
  12. Do you suffer or have you suffered from hypertension?
  □ Yes
  □ No
  13. Do you suffer from diabetes?
  □ Yes
  □ No
  14. Do you suffer or have you suffered from hyperlipidemia?
  □ Yes
  □ No
  15. Have you had cerebral ischemia?
  □ Yes
  □ No
  16. Have you used antidepressants?
  □ Yes
  □ No
  17. Have you suffered from infectious diseases?
  □ Yes
  □ No
  18. Do you have a family history of cognitive impairment and/or dementia?
  □ Yes
  □ No
  19. Do you have neurological diseases in your family?
  □ Yes
  □ No

## Appendix B

Fifteen participants who fulfilled the inclusion criteria were involved in the face validation of the EDQ before conducting the study. After further revising the respective questionnaire based on the feedback obtained, we administred the revised early dementia questionnaire to another 15 subjects and chose the final revised version of the questionnaire for this study. Three of the authors reviewed the questions to ensure that they were consistent with the symptoms of early dementia. We used R software version 4.2.0 for Windows©2009 (Cary, NC, USA) to measure the reliability of the EDQ. The dementia symptoms were classified in 6 groups: concentration, memory, symptoms, emotions, sleep, and physical symptoms. There was a total of 20 questions, where the minimum score was 0 and the maximum score was 60. Cronbach’s alpha coefficient was used to assess the internal test consistency. The estimated Cronbach’s alpha for this questionnaire was 0.792, indicating that the response values for each participant were consistent. A cut-off score of 8 or more for the EDQ was used to indicate that the patient had possible early dementia.
